# Transciptome Analysis of the Gill and Swimbladder of *Takifugu rubripes* by RNA-Seq

**DOI:** 10.1371/journal.pone.0085505

**Published:** 2014-01-16

**Authors:** Jun Cui, Shikai Liu, Bing Zhang, Hongdi Wang, Hongjuan Sun, Shuhui Song, Xuemei Qiu, Yang Liu, Xiuli Wang, Zhiqiang Jiang, Zhanjiang Liu

**Affiliations:** 1 Key Laboratory of Mariculture and Stock Enhancement in North China's Sea, Ministry of Agriculture, Dalian Ocean University, Dalian, China; 2 Beijing Institute of Genomics, Chinese Academy of Sciences, Beijing, China; 3 The Fish Molecular Genetics and Biotechnology Laboratory, Department of Fisheries and Allied Aquacultures and Program of Cell and Molecular Biosciences, Aquatic Genomics Unit, Auburn University, Auburn, Alabama, United States of America; CSIR-Central Drug Research Institute, India

## Abstract

The fish gill, as one of the mucosal barriers, plays an important role in mucosal immune response. The fish swimbladder functions for regulating buoyancy. The fish swimbladder has long been postulated as a homologous organ of the tetrapod lung, but the molecular evidence is scarce. In order to provide new information that is complementary to gill immune genes, initiate new research directions concerning the genetic basis of the gill immune response and understand the molecular function of swimbladder as well as its relationship with lungs, transcriptome analysis of the fugu *Takifugu rubripes* gill and swimbladder was carried out by RNA-Seq. Approximately 55,061,524 and 44,736,850 raw sequence reads from gill and swimbladder were generated, respectively. Gene ontology (GO) and KEGG pathway analysis revealed diverse biological functions and processes. Transcriptome comparison between gill and swimbladder resulted in 3,790 differentially expressed genes, of which 1,520 were up-regulated in the swimbladder while 2,270 were down-regulated. In addition, 406 up regulated isoforms and 296 down regulated isoforms were observed in swimbladder in comparison to gill. By the gene enrichment analysis, the three immune-related pathways and 32 immune-related genes in gill were identified. In swimbladder, five pathways including 43 swimbladder-enriched genes were identified. This work should set the foundation for studying immune-related genes for the mucosal immunity and provide genomic resources to study the relatedness of the fish swimbladder and mammalian lung.

## Introduction

*Takifugu rubripes*, widely distributed in Asia, is one of the most important aquaculture species in China. However, its aquaculture production has been seriously hindered by various infectious diseases which caused major economic losses. To provide insight into mechanisms underlying disease resistance in *T. rubripes*, it is of interest to identify immune related genes, such as in the gill, one of the most important organs for immune response. Mucosal epithelial surfaces act as dynamic interfaces between the external environment and internal milieu [1]. Gill, as one of the mucosal barriers, plays an important role in mucosal immune responses [2].

Several studies on identification of immune-related genes in the gill have been conducted in fish species. A number of differentially expressed immune-related genes including TNFα1, TNFα2, IL-1β2, TGFβ, iNOSa and iNOSb were identified by quantitative real-time PCR in goldfish gill after the *Dactylogyrus intermedius* infection [3]. A partial cDNA sequence of Mx gene, encoding an antiviral effector, was identified in the gill of rare minnow *Gobiocypris rarus* after grass carp reovirus (GCRV) infection. qRT-PCR analysis suggested that expressions of Mx and TLR3 were significantly up-regulated [4]. In addition, many immune-related genes were characterized, such as hepcidin-like and TLR9 in the gill of *Lateolabrax japonicus* and *Ctenopharyngodon idellus*, respectively [5], [6].

In addition to its importance in aquaculture, *T. rubripes* is also widely used as a model system in many scientific fields, especially evolution. For instance, the swimbladder is a specialized organ in teleosts that regulates buoyancy. The homology of swimbladder and the vertebrate lung was reported by the British comparative anatomist Richard Owen as early as in 1846 [7]. Although the homology has been well recognized based on morphological and embryological evidence, molecular evidence is still lacking [8], [9]. Previous research has shown that Hedgehog signaling and Wnt signaling pathways play critical roles in the development of both fish swimbladder and tetrapod lung, the two evolutionary homologous organs [10], [11], [12]. Zheng et al [13] found that genes in cytoskeleton and endoplasmic reticulum were enriched in the zebrafish swimbladder. Several prominent transcription factor genes in the swimbladder including hoxc4a, hoxc6a, hoxc8a and foxf1 were identified and their expressions were confirmed in the development of swimbladder during embryogenesis [13].

Recently, next-generation sequencing-based RNA-Seq analyses have dramatically changed the way to investigate the functional complexity of transcriptome in many organisms [14], [15]. RNA-Seq approach is powerful for unraveling transcriptome complexity, identification of genes, gene associated markers, regulatory non-coding RNAs and for alternative splicing analysis and transcritome profiling [16], [17], [18]. RNA-seq based expression profiling has allowed identification of a large number of immune-related genes in the gill. For instance, Beck et al [19] found that arhamnose-binding lectin (RBL) was dramatically upregulated in the gill of catfish infected with *Flavobacterium columnare*. Sun et al [20] utilized Illumina-based RNA-Seq to examine transcriptome profiling in catfish gill after bath immersion infection and identified a large number of important immune-related genes such as IκBs, BCL-3, TAX1BP1, olfactomedin 4, iNOS2b, IFI44, and VHSV.

In this study, we report the transcriptome analysis of the gill and swimbladder in *T. rubripes* by RNA-Seq analysis. Genes were annotated and enriched in the gill and swimbladder, respectively. Specifically, a set of immune-related genes were enriched in the gill by comparison with the swimbladder. A set of putative homologous genes were identified between the swimbladder and human lung. The transcriptome resources provided, herein, should be valuable for both immune-related studies in the gill and evolutionary analysis of the organs with regard to fish swimbladder and mammalian lung.

## Results

### Distribution of mapped reads throughout the genome

**Figure 1:**
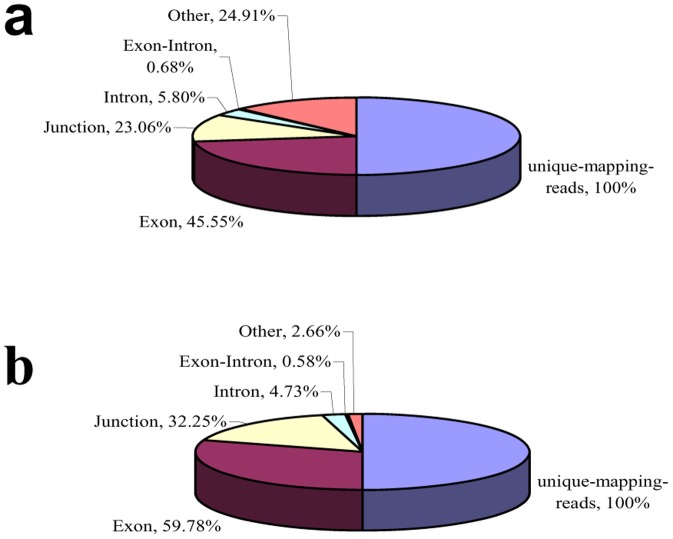
Sequencing reads mapped to various genomic regions. a, gill; b, swimbladder.

Next-generation sequencing was conducted to generate expressed short reads from the gill and swimbladder of *T. rubripes*. As shown in Fig. 1, a total of 27,085,235 and 30,312,181 unique-mapped-reads from 55,061,524 and 44,736,850 raw sequence reads from the gill and swimbladder were obtained, respectively. A number of unique reads were mapped to exons (12,336,515/18,120,867, gill/swimbladder), junctions (6,246,144/9,776,865, gill/swimbladder), introns (1,570,776/1,435,258, gill/swimbladder), exon-intron boundaries (184,135/176,167, gill/swimbladder) and others (6,747,665/803,024, gill/swimbladder). The majority (45.55%/59.78%) of sequence reads were mapped to annotated exons for both gill and swimbladder (Fig. 1).

**Figure 2:**
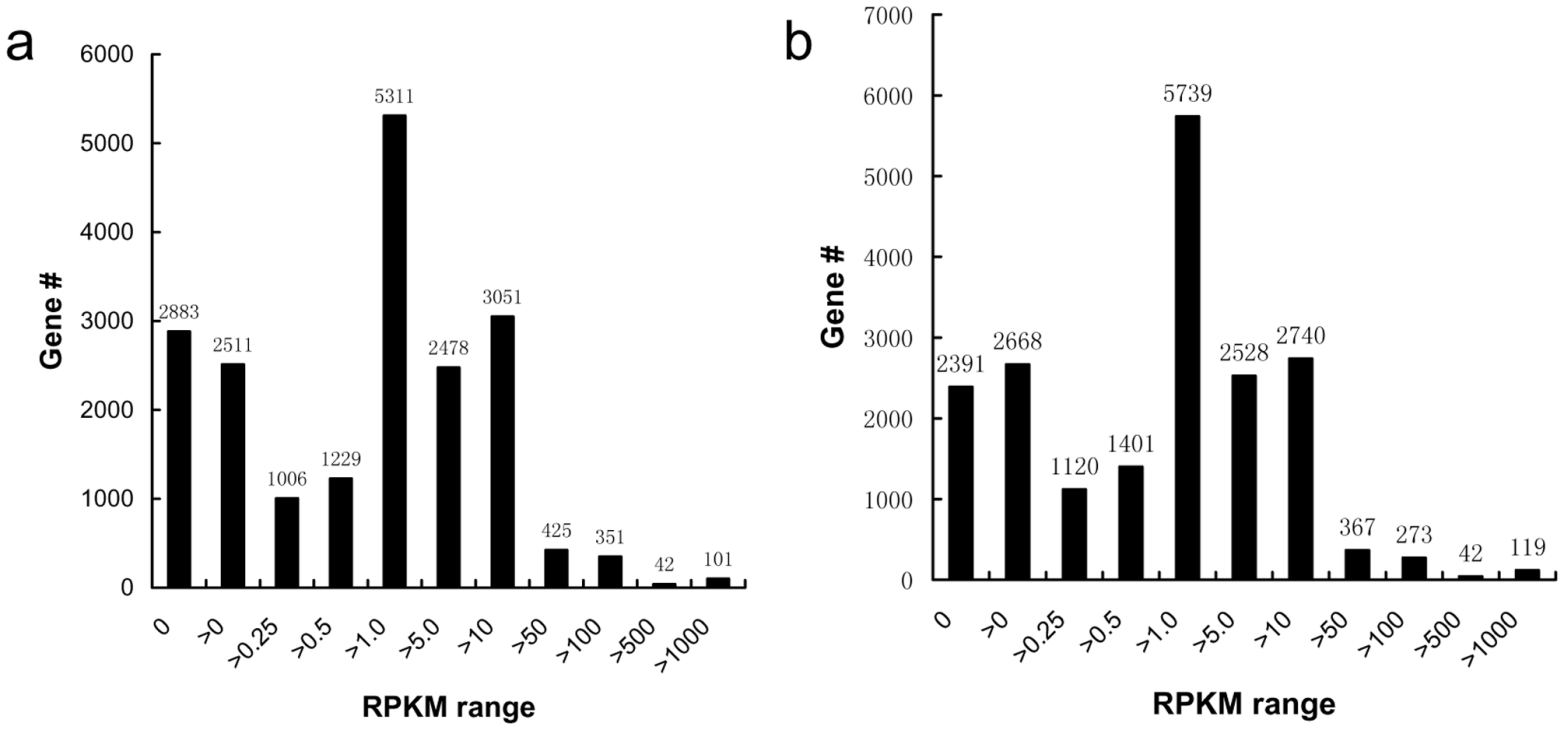
Distribution of genes based on RPKM. a, gill; b, swimbladder.

Expressed short reads were mapped to a total of 19,388 genes (16,836/17,249, gill/swimbladder) based on the fifth fugu *T. rubripes* genome assembly in Ensembl database. With the cutoff values of PRKM setting as 0.05 and 0.08, a total of 15,692 and 15,617 genes were identified as expressed in gill and swimbladder, respectively (Table S1). As shown in Fig. 2, relatively large proportions of genes were highly expressed in both the gill and swimbladder with RPKM ranging from 1.0 to 50. A small number of genes has extremely high expression levels (>500 RPKM). Notably, quite a few genes had no expression or low expression levels (<0.25 RPKM). The hemoglobin beta subunit (ENSTRUG00000016923) had the highest expression levels in both gill and swimbladder, with RPKM of 14,665.38 and 35,972.16, respectively. Similar to previous RNA-Seq studies in zebrafish [13], there were only a few genes which had high expression levels, while most genes were expressed at low levels.

### Functional annotation

Regarding gene annotation, all expressed genes were searched against the Ensembl database. Of the 15,692 expressed genes, 13,111 (83.55%) were annotated in swimbladder, while 12,977 genes were annotated in the gill, accounting for 83.10% of the 15,617 expressed genes.

**Figure 3:**
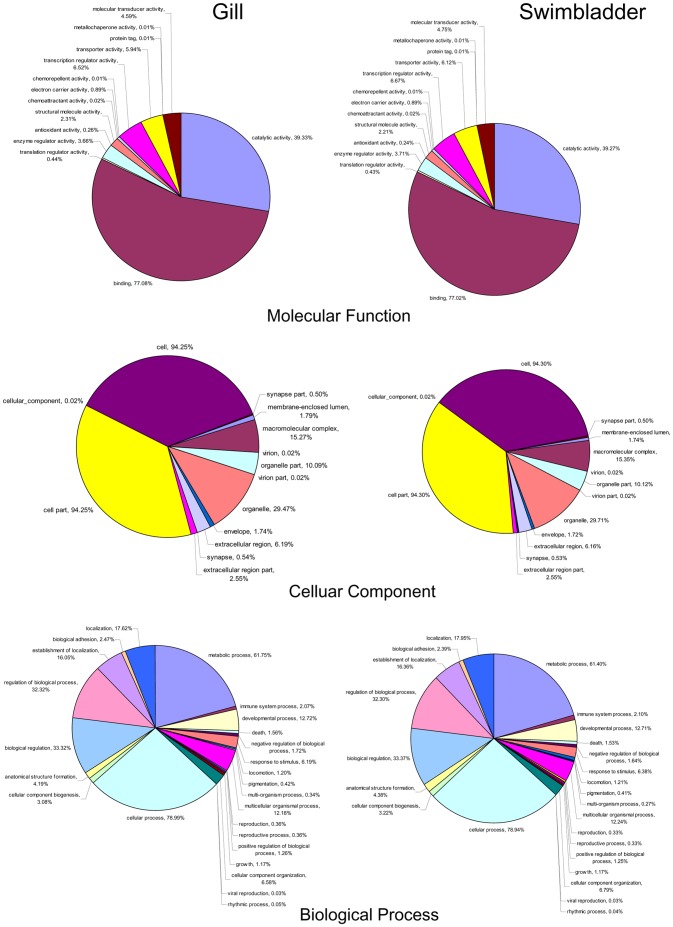
Gene Ontology (GO) (level 2) for the gill and swimbladder transcriptome under Molecular function, Cellular component and Biological process.

Gene ontology (GO) annotation was further performed for the annotated genes in terms of biological process, molecular function and cellular component. Distribution of the genes in different GO categories at level 2 is shown in Fig. 3. In the gill, 7,445, 10,997 and 5,409 genes were assigned with one or more GO terms for biological process, molecular function and cellular component, respectively. The highly represented GO terms were metabolic process and cellular process for biological process, binding and catalytic activity for molecular function, cell part and cell for cellular component. In the swimbladder, 7,294, 11,021 and 7,492 genes were assigned with one or more GO terms for biological process, molecular function and cellular component, respectively. For biological process, genes involved in the metabolic process and cellular process were highly represented. For molecular function, binding was the most represented GO term, followed by catalytic activity. Regarding cellular component, major categories were cell part and cell.

Besides GO analysis, KEGG pathway analysis was also carried out for the annotated genes, which is alternative approach to categorize gene functions with the focus on biochemical pathways. In the gill, a total of 5,582 genes were assigned with one or more KEGG annotation and were mapped to 201 KEGG pathways (Table 1). Of these annotated genes, 47.38% were classified into the cellular processes with majority of which involved in immune system, cell communication and endocrine system. Metabolism pathways including carbohydrate metabolism, lipid metabolism and amino acid metabolism represented 25.40%. Environmental information processing represented 19.88%. The signal transduction was one of the well-represented sub-pathways. In addition, 7.34% were classified into the genetic information processing. In the swimbladder, a total of 5,241 genes were assigned with one or more KEGG annotation and mapped to 201 KEGG pathways (Table 1). Approximately, 47.37%, 25.08%, 20.09% and 7.56% of these annotated genes were classified into cellular processes, metabolism pathways, environmental information processing and genetic information processing, respectively. The most frequently observed KEGG pathways were immune system and signal transduction.

**Table 1:** KEGG biochemical mappings for genes expressed in gill and swimbladder.

KEGG categories	Number of genes	
	gill	swimbladder
Metabolism		
Amino Acid Metabolism	458	451
Biosynthesis of Polyketides and Nonribosomal Peptides	6	6
Biosynthesis of Secondary Metabolites	85	83
Carbohydrate Metabolism	622	615
Energy Metabolism	218	217
Glycan Biosynthesis and Metabolism	192	185
Lipid Metabolism	465	448
Metabolism of Cofactors and Vitamins	224	207
Metabolism of Other Amino Acids	138	138
Nucleotide Metabolism	301	287
Xenobiotics Biodegradation and Metabolism	253	232
Cellular Processes		
Behavior	39	40
Cell Communication	761	762
Cell Growth and Death	521	514
Cell Motility	261	266
Circulatory System	224	218
Development	260	270
Endocrine System	785	756
Immune System	1830	1756
Nervous System	339	334
Sensory System	67	59
Transport and Catabolism	439	443
Environmental Information Processing		
Membrane Transport	44	48
Signal Transduction	1613	1601
Signaling Molecules and Interaction	662	649
Genetic Information Processing		
Folding, Sorting and Degradation	314	314
Replication and Repair	189	188
Transcription	224	222
Translation	129	129

### Identification of the differentially expressed genes and isoforms

Two libraries were constructed in order to identify differential expressed genes between the gill and swimbladder. Transcriptome comparison revealed 3,790 differentially expressed genes (*p*-value < 0.01 and fold-change >2 or <−2), of which 1,520 were up-regulated in the swimbladder while 2,270 genes were down-regulated. Approximately, 74% of these genes (1,206 up-regulated and 1,594 down-regulated genes) were annotated with gene names and descriptions (Table S2).

We defined isoforms as transcripts from the same gene that differ in their transcription as transcripts start site (TSS), coding DNA sequence (CDS), and/or in the 3′untranslated region (3′UTR). The majority of annotated loci produced isoforms found in both swimbladder and gill (about 21,990; Table S3). Of the 21,990 isoforms, 702 isoforms were detected in both samples (p-value < 0.01 and fold-change >2 or <−2). 406 up-regulated isoforms and 296 down-regulated isoforms were observed in swimbladder in comparison to gill.

**Figure 4:**
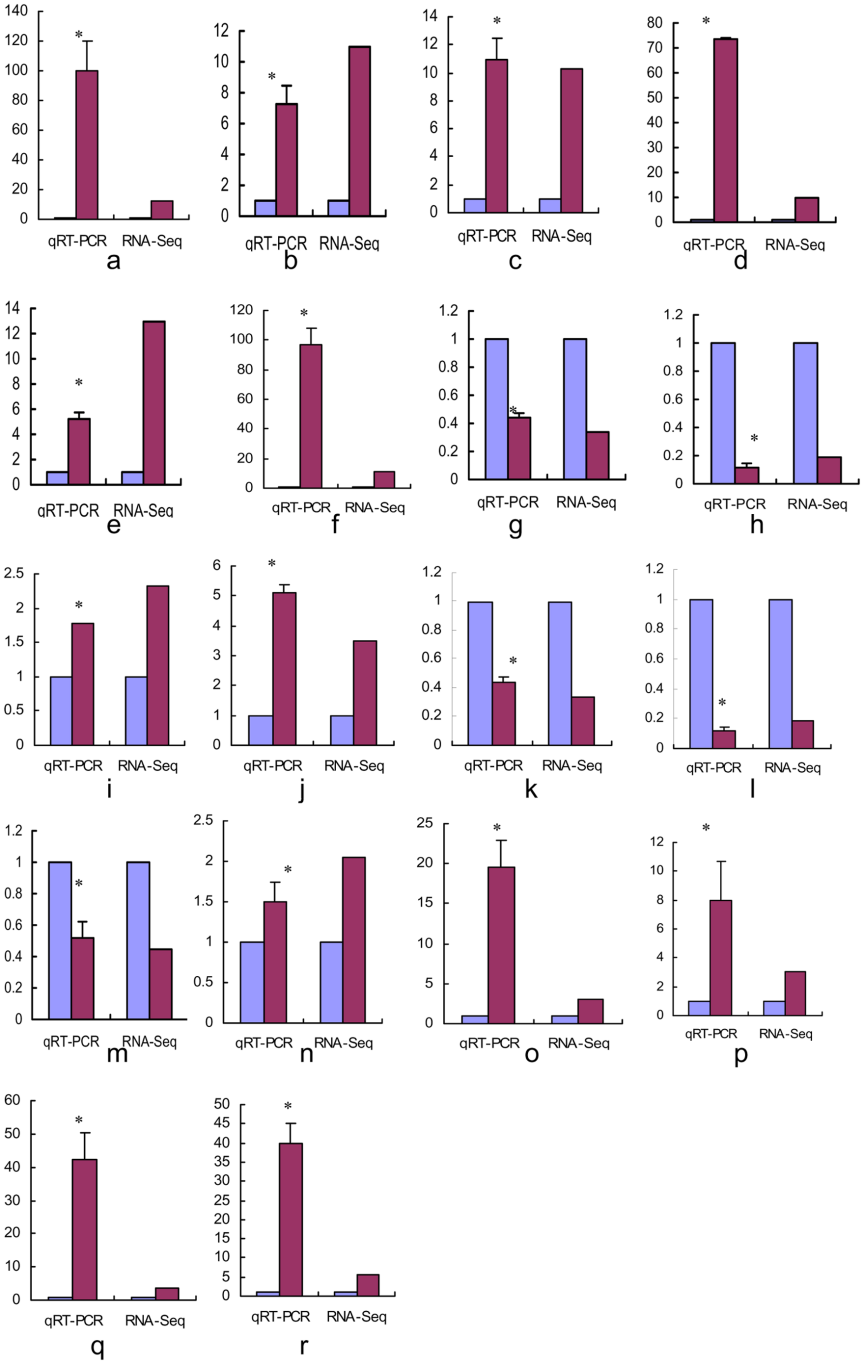
qRT-PCR validation of 18 genes that were differentially expressed between gill and swimbladder. Dark red indicates the tissue of gill and medium slate blue indicates the swimbladder. a, Rh type C glycoprotein2; b, Ras-related protein Rab-19-like; c, Claudin-8-like; d, NAD(P)H dehydrogenase, quinone 1; e, Transmembrane protease serine 11D-like; g, Transmembrane protease serine 9-like; h, Trypsin-like; I, Transcription factor 21-like; j, Minichromosome maintenance complex component 4; k, IL2-inducible T-cell kinase; l, Microphthalmia-associated transcription factor; m, Nuclear receptor subfamily 4, group A, member 2; n, Tetraspanin 11; o, Ankyrin repeat domain-containing protein SOWAHA-like; p, Eukaryotic translation initiation factor 4A2; q, Kelch-like protein 13-like; r, Indian hedgehog protein-like; s, Neuromedin U receptor 1. Asterisk indicates statistical significant level of *P*<0.05.

In order to validate the differentially expressed genes identified by RNA-Seq expression analysis, we randomly selected 18 genes from those with differing expression patterns and from genes of interest based on their functions. Fold changes from qRT-PCR were compared with that from the RNA-Seq expression analysis results. As shown in Fig. 4, the RNA-Seq results were confirmed by the qRT-PCR results, supporting the reliability and accuracy of the RNA-Seq expression analysis.

### Gene set enrichment analysis

By the gene set enrichment analysis, three immune-related pathways (Toll-like receptor signaling pathway; Intestinal immune network for IgA production; Chemokine signaling pathway) and 32 immune-related genes were identified in the gill (Table S4). Five pathways (Wnt signaling pathway; Hedgehog signaling pathway; ECM-receptor interaction; Adherens junction; Tight junction) including 43 swimbladder-enriched-genes were identified in the swimbladder (Table S5).

## Discussion

### Gill immune-related genes

In the gill, 110 annotated genes were immune system process-related genes with most of which involved in antigen processing and presentation, immune effector process, immune response, leukocyte activation, positive regulation of immune system process, regulation of immune system process (Table S6). Immune response was the most represented GO term in immune system process.

**Toll-like receptors** (TLRs) are a class of proteins that play a key role in the innate immune system. They usually expressed in sentinel cells such as macrophages and dendritic cells. They can recognize structurally conserved molecules derived from microbes to activate immune cell responses. TLR genes, such as TLR2/3/5/7/9/21/22, were found in this study. Furthermore, TLR1 and TLR 22 were identified as immune-enriched genes in gill. According to the draft Fugu genome annotation, TLR1/2/3/5/7/8/9/21/22 genes were identified. The TLR4 found in mammals was not identified in the fugu genome, while TLR21 and TLR22 were not found in mammalian genomes [21]. This has been reported in the study of the zebrafish TLR genes [22]. The results suggested that TLR1/2/3/4/5/7/8/9/21/22 genes existed in the ancestral genome common to fish and mammals, and the TLR4 was lost in the fish lineages, while the TLR21 and 22 were lost in the mammalian lineages during evolution.

**Interleukins** (IL) are a group of cytokines that are expressed by white blood cells, and play an important role in transmission information, the activation and regulation of immune cell, the activation, proliferation and differentiation of T and B cell, and the inflammatory response [23], [24]. In this study, five IL genes were identified immune-enriched genes in gill, including IL1/8/10/12/15. In the previous studies, fugu IL genes, such as IL2/6/7/10/12/15/17, were identified and characterized [25]–[31]. The expression of the fugu IL family genes was found to vary in tissues. For instance, IL-10 had a very low level of constitutive expression detected in tissues of healthy fish including liver, kidney, gut and spinal cord, whilst no expression was detected in spleen, gill, brain, gonad and eye. The fugu IL-7 gene was expressed within unstimulated tissues, such as the head kidney, spleen, liver, intestine, gill and muscle. Stimulation of head kidney cells with lipopolysaccharide, polyinosinic polycytidylic acid or phytohaemagglutinin increased the expression. IL-17 family genes were highly expressed in the head kidney and gill. Moreover, expression of IL-17 family genes was significantly up-regulated in the lipopolysaccharide-stimulated head kidney. In addition, PHA stimulation of Fugu kidney cells resulted in a large increase in the expression of fugu IL-6, whereas LPS and Poly I:C resulted in a significant increase within spleen cells.

The expression level of IL-15 was higher because its RPKM was more than the average. IL-15 is a cytokine that possesses a variety of biological functions, including stimulation and maintenance of cellular immune responses [32]. IL-15 stimulates the proliferation of T-lymphocytes and induces production of Natural killer cells. It was found that IL-15 is closely linked to an INPP4B gene like IL-15 from other species, but there was a BCL10 gene located between the IL-15 and INPP4B genes in *Oncorhynchus mykiss* [33]. In *Danio rerio* and *Tetraodon nigroviridis*, analysis by qRT-PCR and in situ hybridization showed that IL15 gene was expressed in lymphoid tissues such as intestine, gills, spleen, pancreas and kidney, suggesting a role in the immune response in fish [34], [35]. Two full-length cDNAs isolated from fugu by RACE–PCR were homologous to known IL-15 genes (IL-15 and IL-15L) [31]. Fugu IL-15 cDNA (Accession no. DQ020256) encoded a peptide of 167 amino acids with a signal peptide of 53 amino acids, while IL-15L cDNA (Acession no. DQ020258) possessed an open reading frame (ORF) for a peptide of 158 amino acids with a signal peptide of 47 amino acids. Expression of the fugu IL-15 gene was found in all the tissues examined including PBL, thymus, head kidney, kidney, spleen, heart, gonad, intestine, skin, gill, brain, muscle and liver. For IL-15L, high expression was detected in spleen, heart, gonad, skin and gill, while weak expression was detected in thymus, head kidney, intestine and liver. No expression was observed in PBL and muscle. The clear differences in expression pattern between the two fugu IL-15 homologues demonstrated that the two genes are likely to play different roles in the fish immune system.

**Chemokines** are a family of small cytokines or signaling proteins secreted by cells. Chemokines have been classified into four main subfamilies: CXC, CC, CX3C and XC. All of these proteins exert their biological effects by interacting with G protein-linked transmembrane receptors called chemokine receptors that are selectively found on the surfaces of their target cells [36]. Two chemokine gene, CCL25, CXCL8 and seven chemokine receptor genes, CCR4, CCR6, CCR7, CCR9 CXCR2,CXCR4 and XCR1 are observed as gill-enriched genes. In the previous study of mammal, chemokine (C-C motif) ligand 25 (CCL25) is a small cytokine of the CC chemokine family that is also known as TECK (Thymus-Expressed Chemokine) [37]. CCR9 and its ligand, CCL25, are the key regulators of thymocyte migration and maturation in normal and inflammatory conditions [38], [39]. Although CCR9 was well studied in mammals, the existence of CCR9 in teleost fish was only reported in a few species such as rainbow trout (*Oncorhynchus mykiss)* [40], zebrafish (*Danio rerio*) and pufferfish (*Takifugu rubripes*) [41], [42]. The zebrafish orthologue of CCL25 was identified and the expression of CCL25 was only detected in the thymus primordia in embryos. In adult fish, transcripts of CCL25 were identified in the thymus, and were also found in the brain and oocytes by in situ hybridization [43]. CXCR4 (also known as fusin) is the receptor for a chemokine known as CXCL12 (or SDF-1),which has a wide cellular distribution, expressing in most immature and mature hematopoietic cell types [44]. In addition, CXCR4 is also found on vascular endothelial cells and neuronal/nerve cells. The sequence of CXCR4 was identified in the rainbow trout. It expressed in head-kidney leukocytes, blood, gill, brain, spleen, and liver [45]. The CXCR4 genes of other fish were identified and studied as well, such as *Cyprinus carpio* [50], *Acipenser ruthenus* [46], *Lamna ditropis* [47], *Danio rerio* [48]. Two zebrafish CXCR4s (CXCR4a and CXCR4b), closely related to mammalian CXCR4, were identified, which were co-expressed in lateral mesoderm and posterior midbrain [49].

### Homologous genes between fugu swimbladder and human lung

**Tight junction** is enriched pathway in swimbladder, which is essential for epithelial morphology and function of swimbladder. Tight junctions serve to form seals between epithelial cells, creating a selectively permeable barrier to intercellular diffusion [13]. Among the swimbladder-enriched genes, the fugu homologs of Cldn (ENSTRUG00000015308) were identified in tight junction. Claudins are transmembrane proteins which act in concert with other transmembrane and peripheral proteins to form the physical basis for tight junction. In zebrafish, cldn4/5/6/7/9 genes were identified in the swimbladder [13]. In human, cldn3/4/5 genes have been found to be co-expressed by type II alveolar epithelial cells [50]. It has been revealed by immunofluorescence staining that cldn4 was increasingly localized to the apical tight junction region, but with lower expression at the lateral region [51]. In contrast, cldn3 and cldn5 are localized exclusively in the apical-most region of the tight junctions. The overexpression of cldn3 decreases solute permeability, whereas cldn5 increases permeability [52].

**Hedgehog signaling and Wnt signaling pathway** play critical roles in development of both tetrapod lung and fish swimbladder, which are the two evolutionary homologous organs. Previous study revealed that down-regulation of Wnt signaling leads to defective swimbladder development [53]. In this study, Twelve and four enriched genes were identified in the two pathway, respectively. Yin et al. [54] demonstrated that gene encoding Wnt signaling member Wnt5b was expressed in different layers of zebrafish swimbladder. Of these genes, Wnt6 and Wnt7B are shared between Hedgehog signaling and Wnt signaling pathway. Wnt proteins play important roles in the growth and morphogenesis of many organs. In lung, Wnt signaling has been proposed to stimulate the proliferation of undifferentiated progenitors [55], [56], to affect proximodistal patterning [57], [58], and to regulate branching morphogenesis. The loss of Wnt7B results in the decreased replication of both epithelium and mesenchyme without grossly perturbing cell differentiation or lung architecture [59]. Wnt7B does interact with known important lung mitogenic pathways and activate an autocrine epithelial and a paracrine mesenchymal canonical Wnt signaling mechanism.

Additionally, transcription factors also play an important role in swimbladder development. **Hox** genes are one of the master regulators of pattern formation during embryogenesis. They function by coordinating cell proliferation, migration, adhesion and differentiation. A total of 11 members of Hox family were identified from our data (Table 2). In a recent zebrafish study, the expression of hoxc4a/6a/8a in developing swimbladder was analyzed. The results indicated that the expression of Hox genes in the swimbladder may not only serve to memorize the positional identity of epithelial cells, but also act as master regulator for adult swimbladder function, such as cellular adhesion and mobility [13]. In human, the Hox genes had distinct expression profiles in the lung. In both human fetal and adult lungs, the most abundantly expressed Hox genes are HoxA5 [60], HoxA10, HoxB2, HoxB9 [61] and HoxB5 [62]. HoxC6 mRNA is detected in both fetal and normal adult lung. In contrast, HoxC8 mRNA is present in the fetal lung [13], [63]. Among these genes, only the homolog of HoxA5 and HoxA10 were expressed in the fugu swimbladder.

**Table 2:** Identification of expressed hox genes in the swimbladder.

Gene ID	Gene name
ENSTRUG00000015981	Homeobox protein Hox-A10a
ENSTRUG00000018252	Homeobox protein Hox-A5a
ENSTRUG00000015974	Homeobox protein Hox-A9a
ENSTRUG00000015327	Homeobox protein Hox-A9b
ENSTRUG00000009707	Homeobox protein Hox-B4a
ENSTRUG00000009487	Homeobox protein Hox-B8a
ENSTRUG00000003635	Homeobox protein Hox-C5a
ENSTRUG00000003727	Homeobox protein Hox-C9a
ENSTRUG00000017520	Homeobox protein Hox-D3a
ENSTRUG00000009562	Homeobox protein Hox-D4b
ENSTRUG00000017521	Homeobox protein Hox-D9a

**Sox** genes encode a family of transcription factors that bind to the minor groove in DNA, and belong to a super-family of genes characterized by a homologous sequence called the HMG (high mobility group) box. A total of 9 Sox genes were found in this study (Table 3). Of these Sox genes, Sox8 and Sox17 were the swimbladder-enriched genes. Sox8 is involved in the regulation of embryonic development and in the determination of the cell fate [64]. Two Sox8 genes (Sox8a and Sox8b) were identified from *Misgurnus anguillicaudatus* and *Paramisgurnus dabryanus* and found essential in the early stage of embryonic development [65], [66]. Sox17 was identified as a key regulator of haemogenic endothelial development. Analysis of Sox17-GFP reporter in mice revealed that Sox17 was expressed in haemogenic endothelium and emerging HSCs suggesting that it was required for HSC development [67]. The Sox17 is an important transcription factor for endodermal cells in *Danio rerio* and is engaged with two other regulatory genes, sox32 and pou5f1 [68]. In addition, it's reported that Sox17 also played an important role in endodermal development and gut tube morphogenesis in the medaka embryo by histology and *in situ* hybridization [69]. To date, at least four members from Sox gene family are known to be involved in lung organogenesis, which are Sox2, Sox9, Sox11 and Sox17 genes [70]–[74]. Sox17 is one of the most extensively studied Sox transcriptional factors in the lung development, which was expressed in respiratory epithelial cells of the fetal lung at embryonic day 18 and was restricted primarily to ciliated cell in the postnatal and adult lung [70], [72], [75], [76]. Sox17 is required for the formation of early endoderm, activating the cell cycle, and reinitiating multipotent progenitor cell behavior in mature lung cells, as well as other development processes such as cardiovascular development, fetal hematopoietic stem cell maintenance, and angiogenesis [70], [75], [77]–[80].

**Table 3:** Identification of sox genes in the swimbladder.

Gene ID	Gene name
ENSTRUG00000014656	Transcription factor Sox6b
ENSTRUG00000009225	Transcription factor Sox8
ENSTRUG00000011266	Transcription factor Sox4
ENSTRUG00000007618	Transcription factor Sox17
ENSTRUG00000000777	Transcription factor Sox18
ENSTRUG00000004861	Transcription factor Sox2
ENSTRUG00000008350	Transcription factor Sox10b
ENSTRUG00000000047	Transcription factor Sox10a
ENSTRUG00000012630	Transcription factor Sox14a

## Materials and Methods

### Ethics statement

This study was approved by the Animal Care and Use committee of Key Laboratory of Mariculture & Stock Enhancement in North China's Sea at Dalian Ocean University. All surgery was performed under sodium pentobarbital anesthesia, and all efforts were made to minimize suffering.

### Sampling of Takifugu rubripes

A total of 45 *Takifugu rubripes* (length 20cm) were sampled from Dalian Tianzheng Industrial Co., Ltd (Dalian China). The gill and swimbladder of these fish were collected and pooled, respectively. Tissues were placed into RNAlater (Ambion), stored at room temperature for 24h, and then moved to −80°C for storage until RNA isolation.

### RNA extraction, library construction and sequencing

Total RNA was extracted from the pooled gill and swimbladder using the TRIzol^R^ Reagent (Invitrogen, CA, USA) following the manufacturer's protocol, respectively. The quantity and quality of total RNA was measured using an Agilent 2100 Bioanalyzer.

Total RNA was sent out for next generation sequencing provided by Beijing Institute of Genomics, Chinese Academy of Sciences. cDNA libraries were constructed from mRNA from gill and swimbladder. cDNA libraries were prepared using the Illumina TruSeq RNA Sample Preparetion Kit (Illumina) according to the TruSeq protocol. After KAPA quantitation and dilution, the libraries were clustered 3 per lane and sequenced on an Illumina HiSeq 2000 instrument with 100 bp paired-end reads.

### Read mapping and functional annotation

Expressed short reads were mapped to the fifth fugu *T. rubripes* genome assembly by BWA program [81], [82]. During the mapping phase, up to five mismatches were allowed. The gene structure information was obtained based on ENSEMBL (http://asia.ensembl.org/index.html) annotation. The expression levels (RPKM, Reads Per Kilobase of exon model per Million mapped reads) for each gene were calculated using uniquely mapped reads by in-house built Perl module according to the equation:
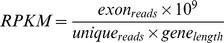



The cutoff value of gene expression was calculated for each sequencing sample, genes with RPKM greater than cutoff value were defined as expressed genes [83]. For functional annotation, the expressed genes were selected and compared against the reference genes from Ensembl database (http://asia.ensembl.org/index.html). Gene Ontology (GO) annotation analysis was performed using Blast2GO, an automated tool for the assignment of GO terms. The annotation result was categorized with respect to Biological Process, Molecular Function, and Cellular Component at level 2. In order to gain an overview of gene pathway networks, KEGG analysis was performed using the online KEGG Automatic Annotation Server (KAAS) (http://www.genome.jp/kegg/kass/). The bi-directional best hit (BBH) method was used to obtain KEGG orthology assignments.

### Transcriptome comparison between gill and swimbladder

The differentially expressed genes between these two samples were identified using DEGseq [84] with *P* value less than 0.001. The fold change was calculated according to the equation:
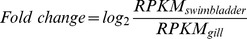



One strength of RNA-Seq is the ability to identify differential expression patterns of gene isoforms [85]. To analyze, raw reads were first aligned to the Fugu reference genome (assembly 5). For this purpose we used the Tophat software, version 2.0.3 [86] with default settings. Aligned reads were assembled by the Cufflinks software (version 2.0.2) [87], [88]. In order to determine which isoforms were differentially expressed within the dataset we used Cuffdiff [86]. Cuffdiff allowed the biological replicate data to be run by Cufflinks as a group, thus enabling identification of differentially expressed isoforms between conditions. Isoforms were considered significant if they met Cuffdiff's requirements to perform a statistical test, had a corrected p-value< 0.01, and an absolute value of the natural log of the fold change > 2.

Genes with absolute fold-change values greater than 2.0 were defined as differentially expressed genes. Eighteen differentially expressed genes were randomly selected for validation using qRT-PCR. The primers were designed using Primer5 software. RNA samples from gill and swimbladder (with fifteen replicate samples each group) were used for qRT-PCR. *β-actin* was used as internal reference gene. The primers of*β-actin* were also shown in Table S5. qRT-PCR was carried out using SYBR® *Premix Ex Taq*™ kit (Takara, Dalian China) and the PCR amplification was quantified according to the manufacturer's instruction. PCR reactions consisted of 1.5 µl first strand cDNA, 7.5 µl SYBR Green (Roche Applied Science), 0.3 µl ROX, 0.6 µl each of 10 µM forward and reverse primers, and 4.5 µl nuclease-free water. qRT-PCR conditions were as follows: 1 cycle at 95°C for 30 sec; 40 cycles at 95°C for 5 sec and 34 sec at 60°C. At the end, a dissociation stage was added: 5 sec at 95°C, 30 sec at 60°C and 30 sec at 95°C.

### Gene set enrichment analysis

There are over 60 enrichment methods and tools to be developed in the past few years [89]. In this study, the DAVID was used to perform gene set enrichment analysis. Gene Ontology Fat categories in the differentially expressed gene sets were analyzed by using DAVID with Fifth Fugu Genome Assembly information as the background and p-values representing a modified Fisher's exact t-test. Unless specifically indicated, the cut-off of p-value is<0.01. KEGG pathway analysis was also performed similarly using DAVID based on Gene Ontology Fat analysis.

The RNA-Seq datasets of *T. rubripes* swimbladder and gill have been deposited to NCBI sequence read archive (SRA). The accession numbers are SRA109280 and SRA109284.

## Supporting Information

Table S1 **The list of of gene annotation information.**
(XLS)

Table S2 **Differentially expressed genes identified by transcriptome comparison between gill and swimbladder.**
(XLS)

Table S3 **Isoforms tracking file.**
(RAR)

Table S4 **The enriched immune-related genes in gill and their KEGG categories.**
(XLS)

Table S5 **The enriched genes in swimbladder and their KEGG categories.**
(XLS)

Table S6 **The genes annotated with the GO term of immune system process.**
(DOC)

Table S7 **Primers used for qRT-PCR validation.**
(DOC)
