# Supplementary material for: Transciptome Analysis of the Gill and Swimbladder of Takifugu rubripes by RNA-Seq
Source: PLoS One. 2014 Jan 16;9(1):e85505. doi: 10.1371/journal.pone.0085505 (PMC3894188; doi:10.1371/journal.pone.0085505)
Supplement: Table S6 — The genes annotated with the GO term of immune system process. (DOC) [file pone.0085505.s006.doc]

Table S6 The genes annotated with the GO term immune system process

| Gene ID | Gene name |
| --- | --- |
| ENSTRUG00000000316 | solute carrier family 11 (proton-coupled divalent metal ion transporters), member 2 |
| ENSTRUG00000000710 | phospholipase C, gamma 1 |
| ENSTRUG00000000792 | chemokine (C-C motif) ligand 25 |
| ENSTRUG00000000827 | matrix metalloproteinase-9 |
| ENSTRUG00000001235 | adenylate kinase 2 |
| ENSTRUG00000001240 | elastin microfibril interfacer 1 |
| ENSTRUG00000001306 | zinc finger protein, multitype 1 |
| ENSTRUG00000001517 | frRunx3/p45 |
| ENSTRUG00000001684 | metastasis associated 1 family, member 3 |
| ENSTRUG00000002063 | transferrin receptor (p90, CD71) |
| ENSTRUG00000002243 | interleukin 15-like |
| ENSTRUG00000002686 | numb homolog (Drosophila)-like |
| ENSTRUG00000003176 | toll-like receptor 2 |
| ENSTRUG00000003617 | Erythropoietin |
| ENSTRUG00000004027 | numb homolog (Drosophila) |
| ENSTRUG00000004103 | Toll-like receptor 21 |
| ENSTRUG00000004107 | Janus kinase 2 |
| ENSTRUG00000004261 | Interleukin 10 homologue |
| ENSTRUG00000004362 | maternal embryonic leucine zipper kinase |
| ENSTRUG00000004413 | dyskeratosis congenita 1, dyskerin |
| ENSTRUG00000004718 | interleukin-12 alpha |
| ENSTRUG00000004759 | toll-like receptor 5 |
| ENSTRUG00000004826 | ribosomal protein L11 |
| ENSTRUG00000004888 | interleukin enhancer binding factor 2, 45kDa |
| ENSTRUG00000005082 | toll-like receptor 3 |
| ENSTRUG00000005329 | v-Ki-ras2 Kirsten rat sarcoma viral oncogene homolog |
| ENSTRUG00000005557 | alanine-glyoxylate aminotransferase 2-like 1 |
| ENSTRUG00000005735 | CD74 molecule, major histocompatibility complex, class II invariant chain |
| ENSTRUG00000005908 | tumor necrosis factor alpha |
| ENSTRUG00000005957 | glutaredoxin 5 |
| ENSTRUG00000006012 | tumor necrosis factor beta |
| ENSTRUG00000006082 | tumor necrosis factor (ligand) superfamily, member 11 |
| ENSTRUG00000006193 | TLR22 |
| ENSTRUG00000006259 | chemokine (C-X-C motif) ligand 12 |
| ENSTRUG00000006262 | zinc finger protein 574 |
| ENSTRUG00000006270 | zinc finger protein 148 |
| ENSTRUG00000006383 | CCAAT/enhancer binding protein (C/EBP), alpha |
| ENSTRUG00000006736 | sorting nexin 5 |
| ENSTRUG00000006788 | interleukin 8 |
| ENSTRUG00000007154 | toll-interleukin 1 receptor domain containing adaptor protein |
| ENSTRUG00000007621 | nucleophosmin (nucleolar phosphoprotein B23, numatrin) |
| ENSTRUG00000007717 | TLR9 |
| ENSTRUG00000007906 | heat shock 70kDa protein 9 (mortalin) |
| ENSTRUG00000008051 | TLR7 |
| ENSTRUG00000008105 | nucleophosmin (nucleolar phosphoprotein B23, numatrin) |
| ENSTRUG00000008218 | T-cell acute lymphocytic leukemia protein 1 |
| ENSTRUG00000008352 | complement component 2 |
| ENSTRUG00000008580 | vascular endothelial growth factor A |
| ENSTRUG00000008735 | Interleukin-6 |
| ENSTRUG00000008959 | pituitary tumor-transforming 1 interacting protein |
| ENSTRUG00000009058 | sprouty homolog 4 (Drosophila) |
| ENSTRUG00000009089 | splicing factor 3a, subunit 3, 60kDa |
| ENSTRUG00000009202 | small nuclear ribonucleoprotein 70kDa (U1) |
| ENSTRUG00000009283 | TATA box-binding protein-like protein 2 |
| ENSTRUG00000009352 | cell division cycle 73, Paf1/RNA polymerase II complex component, homolog (S. cerevisiae) |
| ENSTRUG00000009764 | WD repeat domain 43 |
| ENSTRUG00000009947 | regulator of G-protein signaling 18 |
| ENSTRUG00000009990 | Toll-like receptor |
| ENSTRUG00000010099 | protein kinase C, beta |
| ENSTRUG00000010227 | spectrin, beta, erythrocytic |
| ENSTRUG00000010731 | ectonucleotide pyrophosphatase/phosphodiesterase 2 |
| ENSTRUG00000011157 | colony stimulating factor 3 receptor (granulocyte) |
| ENSTRUG00000011319 | von Hippel-Lindau tumor suppressor-like |
| ENSTRUG00000011323 | chemokine (C-C motif) ligand 25 |
| ENSTRUG00000011336 | solute carrier family 48 (heme transporter), member 1 |
| ENSTRUG00000011858 | troponin T type 2 (cardiac) |
| ENSTRUG00000012176 | nuclear receptor corepressor 2 |
| ENSTRUG00000012333 | ribosomal protein S29 |
| ENSTRUG00000012355 | sushi domain containing 2 |
| ENSTRUG00000012637 | somatomedin B and thrombospondin, type 1 domain containing |
| ENSTRUG00000012748 | LEM domain containing 3 |
| ENSTRUG00000012781 | GATA binding protein 1 (globin transcription factor 1) |
| ENSTRUG00000012896 | single immunoglobulin and toll-interleukin 1 receptor (TIR) domain |
| ENSTRUG00000013092 | serine peptidase inhibitor, Kunitz type 1 |
| ENSTRUG00000013134 | cleavage and polyadenylation specific factor 1, 160kDa |
| ENSTRUG00000013293 | CXXC finger protein 1 |
| ENSTRUG00000013361 | tumor necrosis factor (ligand) superfamily, member 10 |
| ENSTRUG00000013517 | chemokine (C-X-C motif) ligand 12 |
| ENSTRUG00000013543 | Sec23 homolog B (S. cerevisiae) |
| ENSTRUG00000013693 | GATA binding protein 2 |
| ENSTRUG00000013746 | interleukin 15 |
| ENSTRUG00000013770 | ectonucleotide pyrophosphatase/phosphodiesterase 2 |
| ENSTRUG00000014138 | aminolevulinate, delta-, synthase 2 |
| ENSTRUG00000014198 | telomerase reverse transcriptase |
| ENSTRUG00000014431 | tubulointerstitial nephritis antigen-like 1 |
| ENSTRUG00000014535 | bone morphogenetic protein 4 |
| ENSTRUG00000015190 | translocator protein (18kDa) |
| ENSTRUG00000015454 | signal transducer and activator of transcription 5A] |
| ENSTRUG00000015496 | chemokine (C-X-C motif) ligand 14 |
| ENSTRUG00000015663 | prostaglandin E receptor 4 (subtype EP4) |
| ENSTRUG00000015750 | 40S ribosomal protein S7] |
| ENSTRUG00000015820 | tolloid-like 1 |
| ENSTRUG00000016002 | interleukin 1, beta |
| ENSTRUG00000016046 | sterile alpha and TIR motif containing 1 |
| ENSTRUG00000016306 | TNF receptor-associated factor 6, E3 ubiquitin protein ligase |
| ENSTRUG00000016357 | nitric oxide synthase 1 (neuronal) |
| ENSTRUG00000016382 | mediator complex subunit 24 |
| ENSTRUG00000016522 | wingless-type MMTV integration site family, member 5B |
| ENSTRUG00000016704 | solute carrier family 4, anion exchanger, member 1 |
| ENSTRUG00000016842 | transformation/transcription domain-associated protein |
| ENSTRUG00000016902 | TAF3 RNA polymerase II, TATA box binding protein (TBP)-associated factor, 140kDa |
| ENSTRUG00000017059 | LIM domain only 2 (rhombotin-like 1) |
| ENSTRUG00000017125 | interleukin 18 receptor accessory protein |
| ENSTRUG00000017143 | cystic fibrosis transmembrane conductance regulator (ATP-binding cassette sub-family C, member 7) |
| ENSTRUG00000017273 | spleen focus forming virus (SFFV) proviral integration oncogene spi1 |
| ENSTRUG00000017474 | myeloid differentiation primary response protein MyD88 |
| ENSTRUG00000017716 | tumor necrosis factor (ligand) superfamily, member 10 |
| ENSTRUG00000017779 | IKAROS family zinc finger 1 (Ikaros) |
| ENSTRUG00000018174 | myc target 1 |
| ENSTRUG00000018475 | Fas ligand (TNF superfamily, member 6) |
