# Supplementary material for: Transciptome Analysis of the Gill and Swimbladder of Takifugu rubripes by RNA-Seq
Source: PLoS One. 2014 Jan 16;9(1):e85505. doi: 10.1371/journal.pone.0085505 (PMC3894188; doi:10.1371/journal.pone.0085505)
Supplement: Table S7 — Primers used for qRT-PCR validation. (DOC) [file pone.0085505.s007.doc]

**Table S7** PCR primers used for qRT-PCR validation

| No. | Gene ID | Gene name | Primers sequences | |
| --- | --- | --- | --- | --- |
|  |  |  | Sense5'-3' | anti-sense5'-3' |
| a | ENSTRUG00000018092 | 窗体顶端  Rhcg2 , Rh type C glycoprotein2 | GAACGCTTATGTGCGGGTCA | TCGCTGGTTATGTTGTTTGTCTT |
| b | ENSTRUG00000016729 | ras-related protein Rab-19-like | GAGGAGGAACGCCAGGTCA | CGTGCTGCTGGATGTTATGG |
| c | ENSTRUG00000015037 | claudin-8-like | TCTTATTATGACGGGAAGCAGC | GCCAATGACGAGGGCAAC |
| d | ENSTRUG00000011870 | NAD(P)H dehydrogenase, quinone 1 | CCGCAATGCTGGATGGG | CCGTGGTGAGCCCGAAA |
| e | ENSTRUG00000005380 | transmembrane protease serine 11D-like | GTGTCTCATTGCGGAGGGTC | GGGTGGCATACGGCTTGT |
| f | ENSTRUG00000008226 | transmembrane protease serine 9-like | GTGACCACCGGCGACGATA | GCAACGAAACCTGGCTGACT |
| g | ENSTRUG00000002058 | trypsin-like | GGAAGCAACTACCCTGACCG | CGAGGAATCCAGCACAGAACAT |
| h | ENSTRUG00000003919 | transcription factor 21-like | AGCCTTCTCCCGCCTCAA | GGTGGATAAATCCGTTCTCGTAC |
| i | ENSTRUG00000006983 | minichromosome maintenance complex component 4 | GGAGCCTGAACCCAGAAGACA | CCAGACTGTGAGCGTTGTTGC |
| j | ENSTRUG00000004405 | IL2-inducible T-cell kinase | TGCCTTCATGGTTCGGGACT | CTGCTCCTGTTTCTGTCTGTCTG |
| k | ENSTRUG00000012080 | microphthalmia-associated transcription factor | GTATCTCCATCGCATCTTCGG | GGCGTGGCTTTCGTTCAG |
| l | ENSTRUG00000007433 | nuclear receptor subfamily 4, group A, member 2 | AATGGCGGCGGATTGAA | TTGCTGGCGGAGGGACTAG |
| m | ENSTRUG00000015081 | tetraspanin 11 | GGCTGTCTTTGTATTTCTTCTGC | TTCCCTGGCTGGGCGTA |
| n | ENSTRUG00000008047 | ankyrin repeat domain-containing protein SOWAHA-like | ATGGCTTTGACGCAGGAATC | TGGCGACGCTGTTGACG |
| o | ENSTRUG00000000566 | eukaryotic translation initiation factor 4A2 | GCTCGGGCAAATACGGC | CGGAACGCTGGGTTATAGTG |
| p | ENSTRUG00000016000 | kelch-like protein 13-like | GGACGTTCAGCTCAACATAGTGG | AGCGGCGGTTGGGATAG |
| q | ENSTRUG00000012191 | indian hedgehog protein-like | CCAACGCAGACCGCTTCA | CTCCCTGTCGTCAGTGGTTATGT |
| r | ENSTRUG00000006290 | neuromedin U receptor 1 | ACCCAGAGCCGCTTTATCC | TTCACTTCCAGCACTCCTTCC |
| Reference | GenBank: U37499.1 | β-actinⅠ | GCGTGACATCAAGGAGAAGC | TGGGCAACGGAACCTCT |
